# Exploring Antibiotic Resistance Patterns in Isolates from Domestic Animals in Southern Italy

**DOI:** 10.3390/antibiotics15050429

**Published:** 2026-04-24

**Authors:** Tiago Bugarim, Vanessa Maria Bachmann, Marialaura Corrente, Raffaella Sasso, Anna Madio, Marinella Dibari, Vito Martella, Grazia Greco

**Affiliations:** 1 Department of Veterinary Medicine, Universiy of Bari Aldo Moro, 70010 Valenzano, Italy; vanessa.bachmann@uniba.it (V.M.B.); marialaura.corrente@uniba.it (M.C.); grazia.greco@uniba.it (G.G.); 2 Department of Public Health, Experimental and Forensic Medicine, University of Pavia, 27100 Pavia, Italy; 3 Department of Pharmacology and Toxicology, University of Veterinary Medicine, 1078 Budapest, Hungary

**Keywords:** AMR, companion animals, European medicine agency

## Abstract

**Background/Objectives:** Surveillance studies focusing on clinically relevant infections confirm that antimicrobial resistance (AMR) is widespread among bacterial isolates from European households and livestock domestic animals. Due to the shared interface between humans and these animals, as well as an increasing trend in the number of pets per person, the spread of AMR is of concern. **Methods:** In this study, antibiogram reports issued at the bacteriology laboratory of the Department of Veterinary Medicine of the University of Bari from 2020 to 2025 were reviewed and analyzed using descriptive and statistical methods to explore the AMR patterns. **Results:** Two hundred and twenty-eight results were included, comprising 2599 individual tests. A total of 76 molecules across 25 antibiotic classes were tested. Ear swabs were the most common sample type, and *Staphylococcus* spp. and *E. coli* were the most isolated bacteria. Two-thirds of the isolates were susceptible to at least one substance classified by the European Medicine Agency (EMA) as category D. Fairly low non-susceptibility (NS) results were obtained for sulfamethoxazole-trimethoprim (D category), gentamicin (C category), and enrofloxacin (B category). **Conclusions:** An increase in the NS trend was observed over the five-year period. Overall, the results support the need for continuous antibiotic stewardship.

## 1. Introduction

Since the discovery of penicillin and its subsequent use in therapy, selective pressure has been exerted on bacteria, which had to develop defense mechanisms in order to survive [1]. The problem of AMR did not arise with the discovery of the first antibiotic [2]; since then, however, the rapid increase in the use, misuse, and overuse of antibiotics has created conditions for a global health emergency [3].

AMR is regarded as a critical public health problem worldwide, as it is in Europe [4]. It increases the risk of common infections becoming untreatable, as well as medical procedures becoming unsafe [5]. A published report estimates that by 2050 AMR could cause 1.91 million deaths each year, and that a further 8.22 million people will die from illnesses associated with AMR [6]. AMR-bearing bacteria can be acquired through direct [7] and indirect contact with domestic animals [8]. The close relationship between pets and humans, the increasing population trend of dogs and cats in the European Union (EU) (with more than 106 and 120 million, respectively [9]), and their interaction with people, livestock, and their environment [10] emphasizes their role as vectors for bacterial infections or as a source of AMR genes [11,12]. For instance, Menezes et al. [13] have demonstrated that healthy dogs and cats share the same ESBL (Extended-spectrum β-lactamases)/AmpC-producing Enterobacteriaceae strains as their healthy owners.

Surveillance studies focusing on clinically relevant infections further confirm that AMR is widespread among bacterial pathogens isolated from European companion animals [11,12,14,15,16]. AMR is not an isolated veterinary issue but a European public health challenge, requiring harmonized surveillance, prudent antimicrobial use and stewardship strategies aligned with current scientific knowledge.

With the rise in AMR, terms such as ‘multidrug resistant’ have become helpful in describing resistance in different isolates. Although these terms help categorize and compare resistance profiles, they are not consistently used according to the same definitions [17]. Magiorakos et al. [18] standardized the use of these terms and established criteria for different organisms in order to classify them as bearing MDR.

The bacteria *Enterococcus faecium*, *Staphylococcus aureus*, *Klebsiella pneumoniae*, *Acinetobacter baumannii*, *Pseudomonas aeruginosa*, *Enterobacter* spp. and *Escherichia coli* (ESKAPEE) constitute a group of pathogens of particular interest in the context of multidrug resistant infections. ESKAPEE pose a particular treatment challenge as they usually have a higher resistance profile or can easily develop those resistances. As such, the World Health Organization (WHO) has included them in the ‘Global Priority List of Antibiotic-resistant Bacteria to Guide Research, Discovery and Development of New Antibiotics’ [19]. Furthermore, numerous studies have documented the transmission of ESKAPEE bacteria between pets and their owners [20,21,22,23].

The concept that some antibiotics are more valuable than others has led to the categorization of those drugs by the WHO [24]. Additionally, the European Medicines Agency (EMAs) supports the European Commission’s (EC) One Health Action Plan against Antimicrobial Resistance [25]. Within this scope, the agency has been advising the EC on veterinary antibiotic use since 2013 [26]. Moreover, in 2020 EMA has provided a list of antibiotics for veterinary use, based on their importance for human medicine and the risk of AMR. Antibiotic classes are grouped into the following four risk categories: category A—substances to avoid, including antibiotics currently not authorized in food-producing animals and only to be given to pets under exceptional circumstances; category B—restrict substances, which are critically important in human medicine and the use of which in animals should be restricted; category C—substances to be used with caution, covering antibiotics for which alternatives for human use generally exist, but only few alternatives are available in certain veterinary indications; and category D—substances that might be prudently used in animals [27]. Nonetheless, this categorization has been subject to further updates, the last of which was in September 2025, when it was adjusted to align with the list of antimicrobials reserved for human use adopted under Commission Implementing Regulation (EU) 2022/1255. The newest version is available as an infographic, displaying all available classes of antibiotics in their corresponding risk categories, and has been seen as a set of guidelines supporting veterinary surgeons in their treatment choice [28]. However, it has been reported that companion animals are frequently treated using drug classes that are critically important in human medicine [11,29].

There is a gap in information regarding the role of pets as reservoirs and vectors of resistant pathogenic bacteria. Additionally, there is a need for AMR surveillance in other domestic animals to both enrich and keep the geographical database updated. Hence, this review aimed to assess AMR across bacteria isolated at the bacteriology laboratory of the Department of Veterinary Medicine of the University of Bari (DMV) from domestic animals from the region of Puglia, over five years between 2020 and 2025, considering the most important pathogens and antibiotic use stewardship. Our study provides insights into the most common bacterial pathogens and their antibiotic resistance in domestic animals, namely pets and cattle, in order to improve future protocols under the One Health approach.

## 2. Results

From the available reports, 228 bacterial isolates were included in the present study. The samples included were received from 2020 to 2025. Wild animal reports were excluded, so companion animals composed most of the patients, amounting to 79.8% of the total. Ear swabs were the most common type of sample (24.6% of all samples), followed by wound swabs and urine samples, representing 15.8% and 13.6% of the total, respectively. The distribution of isolates per animal species and sample type is presented in Table 1.

**Figure 1:**
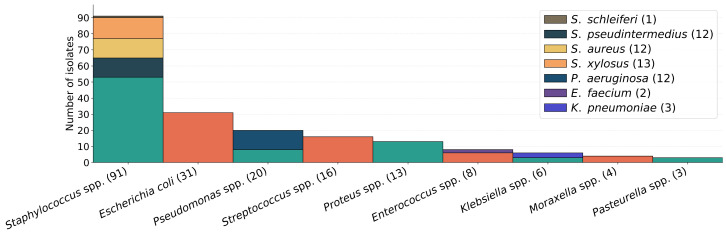
Prevalence of the most common isolates.

Even though the identification method for each isolate was not always recorded, it often included biochemical testing using API^®^ galleries and Polymerase Chain Reaction (PCR) confirmation in some bacterial species. In total, 46 distinct species were isolated, comprised in a total of 24 genera. Most commonly isolated species and genera are presented in the bar chart of Figure 1.

**Figure 2:**
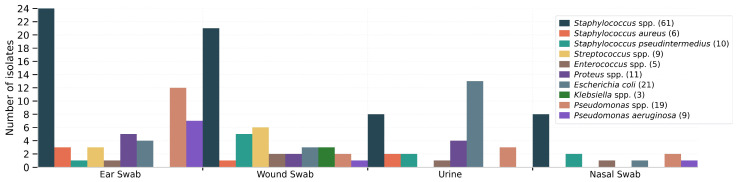
Bar chart with bacterial prevalence per sample type.

In urine samples, *E. coli* was significantly over-represented compared with other bacteria (χ^2^ = 21.49, *p* < 0.001). In nasal swabs, *Staphylococcus* spp. represented the most frequently isolated genus, but without statistical significance (Fisher’s exact test, *p* = 1.00). Figure 2 shows a bar chart with the distribution of the more frequent bacteria by the most common sample type.

**Figure 3:**
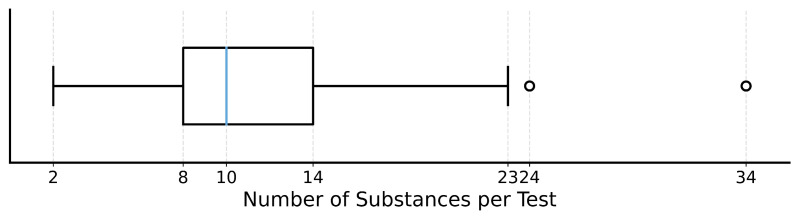
Boxplot with distribution of tested substances per antibiogram.

All the assessed isolates were subjected to the disk diffusion method to evaluate antibiotic resistance. A total of 2599 single antibiotic susceptibility tests were performed, with an average of 11.39 substances per antibiogram, and a median of 10. A boxplot with full information regarding this distribution is presented in Figure 3. The total number of individual substances used throughout the five years was 76.

**Figure 4:**
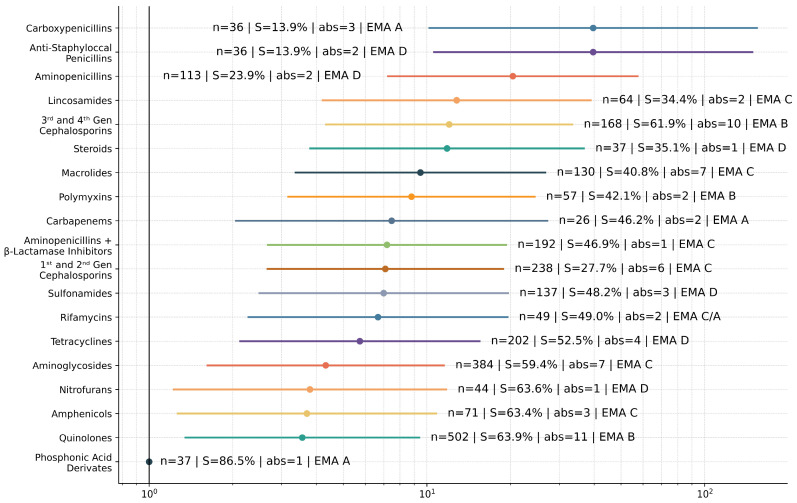
Forest plot with odds ratios of bacterial non-susceptibility against each antibiotic class, along with class information (n = number of times the class was tested, S = overall susceptibility of the class, abs = number of substances in that class, EMA = EMA category).

These substances were grouped into 25 antibiotic classes, displayed along respective NS odds ratios in the forest plot in Figure 4.

Using phosphonic acid derivatives as the reference class, the highest odds of NS were observed for carboxypenicillins [odds ratio (OR) ≈ 39.68, 95% confidence interval (CI) 10.22–154.13, *p* < 0.001] and anti-staphylococcal penicillins (OR ≈ 39.68, 95% CI 10.61–148.34, *p* < 0.001), followed by aminopenicillins (OR ≈ 20.39, 95% CI 7.25–57.36, *p* < 0.001). More modest elevations were seen for tetracyclines (OR ≈ 5.74, 95% CI 2.13–15.46, *p* < 0.001), aminoglycosides (OR ≈ 4.32, 95% CI 1.62–11.51, *p* = 0.003) and quinolones (OR ≈ 3.56, 95% CI 1.35–9.38, *p* = 0.010). Additional information regarding each class is also presented on the plot. Classes containing only one substance were Nitroimidazoles (n = 19, 15.8% S, cat. D), Natural Penicillins (n = 19, 21.1% S, cat. D), Cyclic Polypeptides (n = 13, 7.7% S, cat. D), Monobactams (n = 13, 53.8% S, cat. A), Glycopeptides (n = 11, 63.6% S, cat. A) and Oxazolidinones (n = 1, 100% S, cat. A).

All antibiotic substances tested in more than 20% of the antibiograms are presented in Table 2, along with information regarding their use. For 2599 individually tested substances, susceptibility was confirmed in 1294 (49.8%) cases. Total susceptibility of the antibiotics used in more than 20% of all antibiograms is also presented in Table 2.

*E. faecium* was isolated twice, showing a high resistant profile both times with NS of 93.8% and 88.9%, one being susceptible only to Chloramphenicol and the other only to Gentamicin. There was a total of eight specimens isolated from the genus *Enterococcus* spp. with an average NS of 74.7%. Susceptibility to these isolates was 75% (3/4) to Chloramphenicol and 62.5% (5/8) to Gentamicin. In addition, susceptibility to amoxicillin + clavulanic acid was 66.7% (4/6). One of the overall four isolates that had 0% susceptibility was an *Enterococcus* spp., tested against 23 substances belonging to 15 classes. The other three isolates that showed 100% non-susceptibility were *Micrococcus* spp., *Pseudomonas* spp., and *Staphylococcus* spp., for which nine, eight, and 17 substances were tested, respectively.

**Figure 5:**
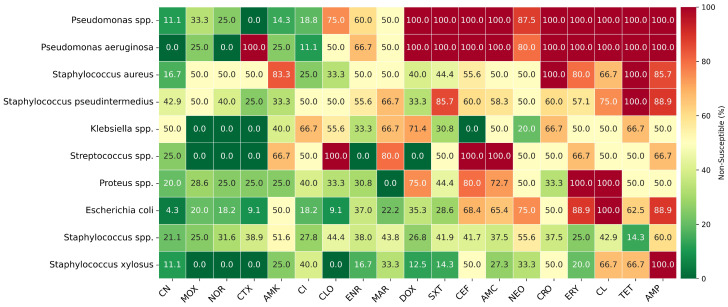
Heatmap of non-susceptibility to most tested antibiotics. AMC = Amoxicillin + Clavulanic Acid, ENR = Enrofloxacin, CN = Gentamicin, SXT = Trimethoprim + Sulfamethoxazole, CEF = Cefalexin, AMP = Ampicillin, DOX = Doxycycline, CI = Ciprofloxacin, AMK = Amikacin, TET = Tetracycline, ERY = Erythromycin, MOX = Moxifloxacin, NEO = Neomycin, CTX = Cefotaxime, CL = Clindamycin, NOR = Norfloxacin, CLO = Chloramphenicol, MAR = Marbofloxacin, CRO = Ceftriaxone.

Figure 5 shows a heatmap of the NS of certain bacteria/genera against the 19 most tested antibiotics.

The *Klebsiella* spp. genus had an overall susceptibility of 43.5% with six isolates. Excluding 4th generation cephalosporins, to which susceptibility was 100% in six tests, average susceptibility to all other β-lactams was 19.7% in seven tests. Average susceptibility to macrolides (in seven tests) and lincosamides (in 12 tests) was 14.3% and 0%, respectively. Tetracyclines were tested 12 times and averaged 62.5% susceptibility, while quinolones achieved 58.3% in eight tests.

Thirty-one isolates were found to be *E. coli* and presented an overall susceptibility of 49.3%. Susceptibility to aminopenicillins was 24.4% in 11 tests and 7.7% to macrolides in 81 tests. For nitrofurans, aminoglycosides, sulphonamides and tetracyclines, the susceptibility was 81.8% in 43 tests, 67.4% in 13 tests, 65.2% in eight tests, and 53.8% in four tests, respectively.

Among isolates tested against gentamicin and amikacin (n = 70), gentamicin demonstrated significantly higher susceptibility results than amikacin (75.7% vs. 55.7%). McNemar’s test confirmed that this difference was statistically significant (χ^2^ = 6.50, *p* = 0.011). Among isolates tested against tetracycline and doxycycline (n = 25), susceptibility to doxycycline exceeded that of tetracycline (40.0% vs. 24.0%), but this difference did not reach statistical significance based on an exact McNemar’s test (*p* = 0.219).

In antibiograms with more than 23 tested substances, susceptibility was 18.3%. MDR was observed in 58.3% (133/228) of the isolates. *E. coli* and *Klebsiella* spp. showed 71.0% (22/31) and 66.7% (4/6) MDR, respectively. Among *S. aureus* isolates, 50% had MDR (6/12), of which 33.3% were methicillin resistant (MRSA) (4/12), based on Magiorakos et al.’s [18] standardized definition. Three out of twelve (25%) isolates of *S. pseudintermedius* were resistant to methicillin. Seven out of the eight *Enterococcus* spp. (including both *E. faecium*) isolates met the criteria for MDR (87.5%%). None of the *P. aeruginosa* isolates showed MDR (0/12).

**Figure 6:**
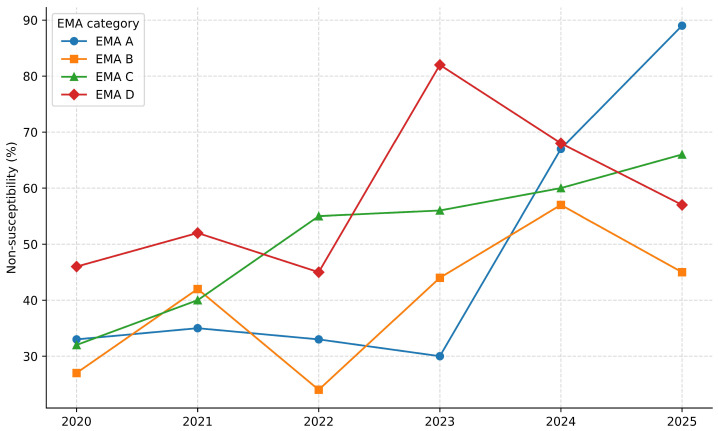
Non-susceptibility trend by EMA category. Each data point is the percentage of NS results in total of tests for that category for that year.

Antibiotics belonging to the four EMA categories were tested. In 45.6% (104/228) of the isolates, one or more substances of category A were tested. Antibiotics from classes B, C, and D were used in 98.2% (224/228), 99.6% (227/228), and 97.4% (222/228) of the total antibiograms, respectively. Total NS to all substances of each category for all antibiograms was 52.3%, 46.4%, 61.2% and 69.1% for categories A, B, C, and D, respectively. NS to one or more substances of the A category when tested was 55.8% (58/104), whereas in 66.7% (148/222) of the times tested, isolates were susceptible to at least one substance of the D category. Figure 6 shows a graph of the NS trend for substances per EMA category. A significant increase in NS was observed for all EMA categories.

For categories A (OR per year = 1.47, 95% CI 1.22–1.78, *p* < 0.001) and B (OR per year = 1.22, 95% CI 1.12–1.32, *p* < 0.001), a significant increase in NS over time was observed. For category C, a strong increasing trend was detected (OR per year = 1.31, 95% CI 1.22–1.32, *p* < 0.001), and for category D, a significant increasing trend in NS was also observed (OR per year = 1.20, 95% CI 1.10–1.32, *p* < 0.001). The Cochran–Armitage test confirmed a statistically significant monotonic increase in NS over time for all EMA categories (EMA A: Z = 4.14, *p* < 0.001; EMA B: Z = 4.83, *p* < 0.001; EMA C: Z = 7.70, *p* < 0.00; EMA D: Z = 3.95, *p* < 0.001). Temporal trends remained consistent across all sensitivity analyses.

## 3. Discussion

We analyzed the AMR patterns of bacterial isolates from companion animal samples submitted to the DVM over a five-year period. Despite the heterogeneous sample type, the distribution aligns with previous works where the majority of samples originated from otitis, wound and urinary tract infection cases [15,30]. The dataset was largely dominated by pets.

The decision to exclude wild animal reports was made to produce a more standardized dataset, as we reasoned the inclusion of a wide range of wild species with very diverse microbiotas would not reflect the most common pathogens found and implicated in the AMR problem and especially the public health concerns associated with it. Therefore, the companion and farm animals included, in our opinion, better represent the human–animal proximity we aimed to target.

The prevalence of specific isolates largely corroborated previous findings [14,15,30,31]. However, we observed a lower incidence of *S. schleiferi* and respiratory-derived isolates, compared to other studies, where these were more frequent. Although *Staphylococcus* spp. was isolated in more than half of respiratory cases, and this is a common finding in the literature [15,31], this association lacked statistical significance, likely due to the limited number of nasal samples. Conversely, the association between *E. coli* and urine samples was statistically significant reflecting its well-established role as a primary urinary pathogen [14,16,31].

While the disk diffusion method has been widely used in bacteriology [32], and this has been the adopted method in our lab, some limitations should be pointed out. For instance, the existence of multiple cutoff values for specific bacteria/antibiotic pairs [33,34], and the potential for human error in inhibition zone reading. Nevertheless, this manual approach allows for flexibility in customizing the antibiotic panel. The median number of substances per test was 10, which is a reasonable amount. As expected, we observed that the number of substances tested increased with the increase in resistance profile. For example, isolates tested against ≥23 substances showed a mean susceptibility rate of only 18.3%, typically representing multidrug-resistant cases.

Overall, 76 individual substances, spanning 25 classes with 1–11 substances per class, were tested. Based on these findings, we suggest that future efforts should focus on standardizing antibiotic panels, particularly by defining a consistent set of representative substances within each class.

To align our findings with regulatory frameworks, antibiotic classes were defined according to the EMA veterinary guidelines [27]. Of note, the rifamycin class comprises rifaximin in EMA category C and rifampicin in EMA category A. We reviewed tests of both substances in our data, and they are grouped together in the classes table, but this had no practical negative impact in the final EMA study; as for that, single substances were used instead of classes. In total, from the 25 present classes, six are categorized as A by EMA, plus rifampicin. Testing for Category A substances remained below 3%, reflecting their restricted use.

For clinical relevance, NS was used to aggregate intermediate (I) and resistant (R) results. While EUCAST generally advises against this grouping [35], it has been used in previous works [30], so it helps comparisons, besides being also defined in the article by Magiorakos et al. [18]. Hence, NS represents the practical viewpoint of all substances not seen as susceptible.

The overall S rate in our data was 49.8%. This is not affected by previous antibiotic administration, as this was an exclusion criterion. Regression analysis supports that decreased susceptibility is not evenly distributed across antimicrobial classes but is instead concentrated within commonly used broad-spectrum antimicrobial classes. Notably, β-lactam classes accounted for four of the five antimicrobial classes with the highest odds of NS, a pattern consistent with both intrinsic resistance mechanisms across bacterial taxa and the effects of selective pressure. Because multiple antimicrobial susceptibility results were recorded per isolate, cluster-robust inference was applied to within-isolate correlation and to avoid underestimation of confidence intervals.

In our dataset, Amoxicillin + Clavulanic Acid was the most frequently tested substance. Despite the overall proportion (46.9%) of isolates categorized as S, a high proportion of NS was observed among the most frequently detected bacterial species, as shown in the heatmap. This finding is consistent with reports describing reduced susceptibility to this combination [30], although another study has documented a higher rate of susceptibility [14].

In previous works, enrofloxacin showed limited effect against *K. pneumoniae*, especially when isolated from cats [14,30], as well as against *Staphylococcus* spp. from cat urine samples [14]. In contrast, in our data enrofloxacin proved fairly effective with an overall S of 61%.

For gentamicin, R above 20% was found only among *Enterococcus* spp. in agreement with previous findings [14,15]. However, markedly higher proportions of R (52.2–78.6%) across all isolates have been reported elsewhere [30].

For Trimethoprim + Sulfamethoxazole (SXT), in one study, only about 30% of *Proteus* spp. from urine samples were resistant [14]. Studies including a broader range of specimen types like ours have reported higher resistance, up to 75% for *E. coli* and *P. mirabilis,* and approximately 50% for other taxa [30]. These results are in line with both our data and another paper where resistance varied between 15 and 50% for every isolate except *Pseudomonas* spp., which reached 90% R [15].

Doxycycline has been reported to exhibit resistance rates ranging from 20% to 50% across most isolates [14,15], a finding consistent with our results, which showed a total susceptibility of 60.4%. In our sample, tetracycline showed a NS of 59.7%. High resistance to tetracycline in *Staphylococcus* spp., *Pseudomonas* spp., and *Klebsiella* spp. isolates has been highlighted in another work [14]. Overall, tetracycline showed limited activity against the most prevalent pathogens, whereas doxycycline remained comparatively effective. Reduced efficacy for the latter was observed primarily against *Proteus* spp. and *Pseudomonas* spp. Notably, *Proteus* spp. showed only a moderate resistance profile. SXT was effective in 65.6% of these isolates and aminoglycoside NS rate did not exceed 25%.

*Pseudomonas* spp. and particularly *P. aeruginosa* showed the highest overall resistance levels, in agreement with a previous article [15]. These bacteria are inherently resistant to cephalosporins and SXT, as seen in our tests, but they also showed high NS to tetracyclines and macrolides, as reported before [14], and to lincosamides, unlike that same report. While certain other reports describe high resistance to aminoglycosides from this genus [15], others, including the present study, identified this class as the most effective therapeutic option [14].

*Staphylococcus* spp. showed a moderate resistance profile, with NS to most commonly tested substances ranging from 14% to 67%. Notably, *S. aureus* and *S. pseudintermedius* had considerably higher resistance profiles, with 100% NS to tetracycline. By contrast, doxycycline had robust susceptibility against this genus, with maximum NS of 40% for *S. aureus*. Even though *S. aureus* showed high NS to amikacin, and previous reports showed mixed results from *Staphylococcus* spp. to aminoglycosides [14,15,30], gentamicin still showed good activity across all isolates in this genus, in our data.

Regarding *Enterococcus* spp., though one study reports aminoglycoside resistance exceeding 50% [15], our data showed high efficacy from gentamicin. Furthermore, our findings for chloramphenicol corroborate prior reports of high amphenicol susceptibility (e.g., florfenicol 97.8%; chloramphenicol 93.6%) in this genus [14].

Finally, while one previous study found that approximately 90% of feline urinary *Klebsiella* spp. were resistant to tetracycline [14], non-susceptibility in our study was lower (50%). Doxycycline NS did not exceed 33.3%, suggesting that tetracyclines may remain viable therapeutic options in selected clinical cases. Resistance profiles of *E. coli* reported in the literature exhibit significant variability. In the present study, *E. coli* isolates showed high NS to ampicillin and moderately high NS to tetracycline and amikacin. While previous reports have cited tetracycline NS rates as low as 30% [15], other studies have reported levels reaching 81–90% [30]. Within the tetracycline subclass, our data indicated that doxycycline possessed greater activity (35.3% NS) than tetracycline, suggesting a trend toward improved susceptibility. Regarding aminoglycosides, while complete resistance to amikacin has been documented elsewhere [14], gentamicin remained the most active agent tested in both our data and prior reports [14,16].

Consistent with the established literature [30], *Klebsiella* spp. and *E. coli* showed the highest MDR rates. In the reviewed data 66.7% (4/6) of Klebsiella spp. and 71.0% (22/31) of E. coli isolates presented MDR, aligning with the high resistance profiles reported. However, for *E. coli*, SXT seems to represent a good first choice, as the data showed 71.4% S with this antibiotic. In addition, gentamicin was effective against all but one isolate, which showed intermediate susceptibility. Notably, 87.5% (7/8) of Enterococcus spp. isolates met the criteria for MDR. However, gentamicin and amoxicillin–clavulanic acid retained moderate efficacy against these isolates, though the latter’s interpretation is constrained by the limited number of samples (60% susceptibility; n = 5).

Although clinical guidelines often extrapolate resistance from a single representative agent to an entire antimicrobial class [18], our findings challenge this assumption. We observed discordant susceptibility profiles within the tetracycline and aminoglycoside classes. Paired susceptibility analyses provide a more rigorous comparison of antimicrobial effectiveness. While doxycycline demonstrated higher susceptibility than tetracycline, using this approach, the difference was not statistically significant, likely reflecting limited statistical power due to the small number of paired isolates. By contrast, gentamicin was found to be significantly more active than amikacin, suggesting a meaningful difference in clinical effectiveness. These differences in susceptibility among aminoglycosides are often driven by distinct aminoglycoside-modifying enzymes (AMEs). Enzymes such as acetyltransferases and phosphotransferases exhibit substrate specificity; consequently, an isolate may harbor AMEs that inactivate gentamicin but leave amikacin unaffected, or vice versa [36,37]. Thus, such same-isolate discordance between aminoglycosides suggests resistance by these AMEs and highlights that agents within this class are not fully interchangeable.

A key study on antibiotic prescribing practices among veterinary surgeons in the south of Italy [29] reported the widespread use of critically important antimicrobials for human health, with most prescribed antibiotics in category C, followed by category B and the lower amount from category D. However, reviews of antibiotic use in accordance with EMA antibiotic categorization (or other entities) are not vast in the literature. When antibiotic use reports exist, they usually either present antibiotic use in quantities (mass) [38,39] or the data are based on questionnaires [40]. Moreover, reviews with AMR data, like ours, do not necessarily correlate directly with antibiotic use, as these data are based only on the antimicrobial resistance studied and/or reports delivered to the clinicians. The follow-up on which antibiotics are, in fact, used by the clinician is not routinely done by our lab. This follow-up or other concrete antibiotic use surveillance methods would be useful for studies on compliance with antibiotic use guidelines, such as the EMA infographic on veterinary antibiotic use. One study within this line of thinking found that almost two-thirds of antibiotics used were from the critically important antimicrobial of high priority or critically important antimicrobial of highest priority classes according to WHO [11,41]. As another example, when inquired about antibiotic use for respiratory disease in cattle, 70% of veterinary surgeons from 25 European countries stated their first choice was a substance in category C or above, while only 23% replied tetracyclines (D category) [40]. For comparison, in our data, all isolates from respiratory cases in cattle (5) were susceptible to at least one substance from the tetracycline class (D category). Beyond that, 66.7% of the times tested, isolates were susceptible to at least one substance in category D. Thus, according to our data, there is no reason to blindly choose a substance from categories C, B or A instead of a substance in category D in these situations. This aligns with the idea that antibiotic stewardship and clinical success can be compatible.

In almost all antibiograms, substances of categories B, C, and D are present, whereas category A substances were used in almost half of the tests. This is possibly because their use is almost always included in any given surveillance project and is, instead, avoided when not strictly necessary for clinical purposes. With these data and the statistical analysis carried out, we can appreciate the results in the trend graph presented before, which shows a clear increase in NS values over the years. Across all EMA categories, the odds of a test being NS increased significantly with calendar year, even after accounting for unequal and/or low numbers of tests per year and per category. The results of the Cochran–Armitage test also corroborate the findings from the logistic regression and support the presence of a real temporal trend rather than an artifact of changing sample sizes. Importantly, this trend was also evident for category A and B antimicrobials, which are considered critically important or subject to restriction. The strongest temporal increase was observed for category C antimicrobials, which are commonly used as second-line agents. This pattern may reflect sustained selective pressure associated with their clinical use. Together with a significant upward trend also detected for category D substances, this might point to a gradual erosion of susceptibility to first-line treatment options. The trend observed for category A should be interpreted with caution, as fewer substances and tests contribute to this category. Nevertheless, the detection of a significant increase in non-susceptibility among critically important, and first- and second-line antimicrobials are of particular concern and highlights the need for continued antimicrobial stewardship.

Regarding infection type and host species distribution, care should be taken while drawing any conclusions as these results are not expected to reflect either the true epidemiological distribution of infections across animal species. Although subjecting an infection case to sampling for bacterial identification and antibiogram is largely advised in accordance with antibiotic stewardship [42,43], it is well-recognized that this might not be done routinely [44,45] and this decision is always up to the clinician and might not be done routinely for several different reasons [44,46].

Bacterial identification, at the time of sampling, was done through phenotypical methods, although some specific cases were always subjected to PCR confirmation, for instance, Methicillin Resistant Staphylococci (MRS) isolates. While phenotypical approaches remain widely used, they present some limitations especially concerning the time needed and discrimination between closely related or uncommon species [47]. As a result, only genus-level identification was possible in some of the cases. These isolates were nonetheless included, as the main focus of our study was AMR profiles, and these isolates still yielded valid antibiogram results. In addition, grouping these isolates into genera along with the remaining corresponding isolates further strengthened the results.

## 4. Materials and Methods

### 4.1. Sampling

Samples received at the DVM were sent by veterinary surgeons working at the Veterinary Hospital of the University of Bari, private working veterinary surgeons, private veterinary clinics, and other laboratories in the region of Apulia, Southern Italy. Upon receival, samples were given an ID number and data regarding the case was collected, namely the sample type, the animal species, age, and breed, owner data, case anamnesis, and history, including previous antibiotic administration for bacteriology samples. Samples included swabs from the suspected infection site and different tissues or matrices such as exudates, milk, blood, semen, urine, and organs collected during necropsy.

### 4.2. Bacterial Identification

The samples were cultured on the day of arrival for bacterial identification, according to an in-house defined protocol for each specific case. Briefly, and apart from blood samples which were processed following their specific protocol, these samples were cultured in an aerobic, anaerobic or microaerophilic environment depending on the sampling anatomical site. Three Petri dishes containing different media were cultured—tryptic soy agar supplemented with 5% sheep’s blood (Liofilchem^®^ Ref. 11037), Mannitol Salt Agar (Liofilchem^®^ Ref. 610029) and MacConkey agar (Liofilchem^®^ Ref. 610028) (Liofilchem, Roseto degli Abruzzi, Italy). After 24–48 h of incubation at 37.0 °C, in one of the three previously mentioned environments, suspected single colonies were used for identification through Gram staining, possibly further culture in specialized selective media, biochemical identification with catalase and oxidase tests, and API^®^ galleries (bioMérieux Italia S.P.A., Grassina, Italy). In some cases, molecular methods were employed, such as Polymerase Chain Reaction (PCR), to aid or confirm identity. Antibiotic susceptibility testing was performed after definitive identification or, sometimes, right away when pure colonies were isolated.

### 4.3. Antibiotic Susceptibility Testing

Antibiotic susceptibility testing was performed following the Kirby-Bauer test [32]. Up to 8 antibiotic-impregnated disks (Liofilchem^®^) were used in each Petri dish. Antibiotics were selected considering the patient species, infection location, bacterial species, available commercial formulations, and EMA categories; however, more substances were often added within the scope of research and/or epidemiological surveillance. The results were interpreted using the manufacturer’s provided guidelines [48] (based on both CLSI [33] and EUCAST [34]), and a clinical report with the results was issued. Methicillin resistance by staphylococci was confirmed by PCR.

All the available antibiogram reports from the bacteriology lab at DVM from 2020 until 2025 were reviewed. For the present study, antibiograms from wild animal cases were excluded, meaning that the included species comprised only companion animals (dogs and cats) and farm animals (horses, cattle, sheep, and goats). Reports missing any crucial information, such as animal species or incomplete antibiogram tables, were also excluded, as well as cases with a recent antibiotic administration.

### 4.4. Data Processing

Upon review, the information was gathered and organized using Microsoft Excel 365 (Microsoft Corp., Redmond, WA, USA). This information included, for each sample, the ID number, date of antibiogram reading, animal species, sample type, bacterial species identified, antibiogram method, antibiotics used, and antibiogram results. Antibiogram results were presented as susceptible (S), intermediate (I), and resistant (R). For analysis purposes, another result was later calculated: non-susceptibility (NS), i.e., the sum of R and I results.

Antibiotics were grouped in 26 classes to align with the classes categorized by EMA’s latest infographic [19]. For the study on MDR, the cut-off was set as resistance to at least one substance in three or more of the defined antibiotic classes. However, specifically for the study on MDR of *S. aureus*, *Enterococcus* spp. and *Pseudomonas aeruginosa*, the classes were set as proposed by Magiorakos et al. [18] for each studied organism. Bacteria were grouped by genus to obtain some of the presented data.

Both descriptive and statistical studies were performed on the data. For the statistical analysis, IBM SPSS Statistics, version 29 (IBM Corp., Armonk, NY, USA) was used. Differences in bacterial distribution within specific sample types were assessed using chi-square or Fisher’s exact tests, as appropriate. Tests were two-sided, with *p*-values < 0.05 considered statistically significant. To quantify differences in NS between antimicrobial classes, a logistic regression model was fitted with NS as the binary outcome and antimicrobial class as the explanatory variable. Because multiple antimicrobial results were recorded per isolate, cluster-robust standard errors were used with clustering at the isolate level. Odds ratios (ORs) with 95% confidence intervals (CIs) were reported relative to the class with the lowest NS rate among classes with adequate representation (≥20 tests). Classes with fewer than 20 tests were excluded from regression modeling. For paired comparisons with enough discordant observations, McNemar’s chi-square test with continuity correction was applied (gentamicin vs. amikacin), whereas exact McNemar’s tests were used for comparisons with small sample sizes and low numbers of discordant pairs (tetracycline vs. doxycycline). Analyses were restricted to isolates tested against both agents, and susceptibility was dichotomized as S versus NS.

For the EMA temporal trend, NS was assessed using isolate-level antimicrobial susceptibility test results. Each single substance test result was treated as a single observation at that time point. To evaluate temporal trends while accounting for unequal numbers of tests across years and EMA categories, logistic regression models were fitted separately for each EMA category, with NS as the binary outcome and calendar year as a continuous predictor. Results are reported as ORs per year with 95% CI. As a confirmatory analysis, Cochran–Armitage tests for trend were performed for each EMA category to assess monotonic changes in NS proportions over time. All tests were two-sided, and *p*-values < 0.05 were considered statistically significant. To confirm the observed temporal trend, sensitivity analysis was conducted. Testing was repeated after excluding isolates classified as intermediate, restricting the outcome to resistant versus susceptible only; calendar years were aggregated into multi-year periods to reduce potential instability arising from low yearly sample sizes; calendar years with very low numbers of isolates were excluded; restriction to antimicrobials with more frequent testing, excluding rarely tested substances; and only with a core antimicrobial panel tested consistently across all study years and spanning all EMA categories. All sensitivity analyses were performed using the same logistic regression and Cochran–Armitage trend testing framework as the primary analysis, thus stressing the conclusion.

## 5. Conclusions

The main positive takeaway is that two-thirds of all isolates were susceptible to at least one substance in EMA category D. This supports the idea that most cases can be treated with lower-risk antibiotics complying with antibiotic stewardship and, as such, we strongly advise testing with these substances. We were able to identify at least one substance in the first three EMA categories with fairly low non-susceptibility results: SXT as a first choice (D category), then gentamicin (C category), and as a last resort, enrofloxacin (B category).

Against what is commonly accepted, we found some discrepancies regarding intra-class resistance. This was largely noticed between doxycycline and tetracycline, although without statistical significance. In contrast, between aminoglycosides and among the same isolates, gentamicin was significantly more effective than amikacin. Thus, we advise testing more than one substance per class, as this might be especially helpful in high-resistance isolates.

*Pseudomonas* spp. had the highest resistance profile in our data, and, given the limited lower category options, aminoglycosides appear to be the best option to test for. For *E. coli*, a species with diverse resistance profiles reported, and *Staphylococcus* spp. we have seen satisfactory results with two first choice antibiotics: SXT and gentamicin. For *Staphylococcus* spp., one major pathogen in companion animals, doxycycline and gentamicin had the best susceptibility results.

The main negative takeaway is the observed increase in non-susceptibility over the years, emphasizing the need for continued antimicrobial stewardship.

Above all, we hope this extensive review of the data from our lab will provide some insights for clinicians and other labs for further reference.

## Figures and Tables

**Table 1:** Sample distribution per sample type and animal species.

		Animal Species							
		Canine	Feline	Bovine	Equine	Caprine	Ovine	Total	Total %
Sample Type	Ear Swab	42	11	-	3	-	-	56	24.6%
	Wound Swab	30	10	2	4	3	-	49	21.5%
	Urine	15	16	-	-	-	-	31	13.6%
	Vaginal Swab	20	-	-	1	-	-	21	9.2%
	Organ	9	5	3	-	-	2	19	8.3%
	Nasal Swab	3	5	5	2	-	-	15	6.6%
	Milk	2	-	11	-	-	-	13	5.7%
	Eye Swab	3	1	5	2	-	-	11	4.8%
	Blood	-	5	-	-	-	-	5	2.2%
	Oral Swab	2	-	-	2	-	-	4	1.8%
	Rectal Swab	2	-	-	1	-	-	3	1.3%
	Semen	1	-	-	-	-	-	1	0.4%
	Total	129	53	26	15	3	2	228	100.0%
	Total %	56.6%	23.3%	11.4%	6.6%	1.3%	0.9%	100.0%	

**Table 2:** Substances tested in more than 20% of antibiograms.

Substance	t	% AST	S	Substance	t	% AST	S
AMC	192	84.2%	46.9%	ERY	65	28.5%	41.5%
ENR	182	79.8%	61.0%	MOX	62	27.2%	72.6%
CN	161	70.6%	82.0%	NEO	59	25.9%	33.9%
SXT	117	51.3%	53.8%	CTX	56	24.6%	46.4%
CEF	108	47.4%	43.5%	CL	55	24.1%	36.4%
AMP	101	44.3%	25.7%	NOR	55	24.1%	70.9%
DOX	96	42.1%	60.4%	CLO	52	22.8%	61.5%
CI	89	39.0%	73.0%	MAR	49	21.5%	61.2%
AMK	85	37.3%	52.9%	CRO	48	21.1%	62.5%
TET	67	29.4%	40.3%				

t = times tested, % AST = presence in antibiograms, S = susceptibility, AMC = Amoxicillin + Clavulanic Acid, ENR = Enrofloxacin, CN = Gentamicin, SXT = Trimethoprim + Sulfamethoxazole, CEF = Cefalexin, AMP = Ampicillin, DOX = Doxycycline, CI = Ciprofloxacin, AMK = Amikacin, TET = Tetracycline, ERY = Erythromycin, MOX = Moxifloxacin, NEO = Neomycin, CTX = Cefotaxime, CL = Clindamycin, NOR = Norfloxacin, CLO = Chloramphenicol, MAR = Marbofloxacin, CRO = Ceftriaxone.

## Data Availability

All data created for this review are presented in the paper.

## Abbreviations

The following abbreviations are used in this manuscript:

AMR	Antimicrobial resistance
ESKAPEE	*Enterococcus faecium*, *Staphylococcus aureus*, *Klebsiella pneumoniae*, *Acinetobacter baumannii*, *Pseudomonas aeruginosa*, *Enterobacter* spp. and *Escherichia coli*
WHO	World Health Organization
MDR	Multidrug resistance
EMA	European Medicines Agency
EC	European Commission
DMV	Department of Veterinary Medicine of Bari University
PCR	Polymerase Chain Reaction
NS	Non-susceptibility
ESBL	Extended-spectrum β-lactamases
OR	Odds-ratio
CI	Confidence interval
S	Susceptibility
CLSI	Clinical and Laboratory Standards Institute
EUCAST	European Committee on Antimicrobial Susceptibility Testing
I	Intermediate
R	Resistant
